# The Elaborated Environmental Stress Hypothesis as a Framework for Understanding the Association Between Motor Skills and Internalizing Problems: A Mini-Review

**DOI:** 10.3389/fpsyg.2016.00239

**Published:** 2016-02-23

**Authors:** Vincent O. Mancini, Daniela Rigoli, John Cairney, Lynne D. Roberts, Jan P. Piek

**Affiliations:** ^1^School of Psychology and Speech Pathology, Curtin UniversityPerth, WA, Australia; ^2^Department of Family Medicine, McMaster UniversityHamilton, ON, Canada

**Keywords:** motor coordination, motor skills, motor proficiency, environmental stress, internalizing problems, anxiety, depression

## Abstract

Poor motor skills have been shown to be associated with a range of psychosocial issues, including internalizing problems (anxiety and depression). While well-documented empirically, our understanding of why this relationship occurs remains theoretically underdeveloped. The Elaborated Environmental Stress Hypothesis by Cairney et al. (2013) provides a promising framework that seeks to explain the association between motor skills and internalizing problems, specifically in children with developmental coordination disorder (DCD). The framework posits that poor motor skills predispose the development of internalizing problems via interactions with intermediary environmental stressors. At the time the model was proposed, limited direct evidence was available to support or refute the framework. Several studies and developments related to the framework have since been published. This mini-review seeks to provide an up-to-date overview of recent developments related to the Elaborated Environmental Stress Hypothesis. We briefly discuss the past research that led to its development, before moving to studies that have investigated the framework since it was proposed. While originally developed within the context of DCD in childhood, recent developments have found support for the model in community samples. Through the reviewed literature, this article provides support for the Elaborated Environmental Stress Hypothesis as a promising theoretical framework that explains the psychosocial correlates across the broader spectrum of motor ability. However, given its recent conceptualization, ongoing evaluation of the Elaborated Environmental Stress Hypothesis is recommended.

There is growing recognition that motor skills have a significant role in psychosocial development. The ability to make accurate and coordinated age-appropriate movements enables opportunities for the optimal development of psychosocial wellbeing. Indeed, studies have shown that poor motor skills are associated with a range of negative psychosocial outcomes. More recently, studies have investigated the psychosocial implications of motor skills in both clinical and non-clinical populations; while early studies often focused on the physical deficits experienced by children diagnosed with developmental coordination disorder (DCD). DCD is a neurodevelopmental disorder characterized by poor motor skills that are unrelated to other physical and/or intellectual impairments (American Psychiatric Association, 2013). The prevalence of DCD is estimated to be between 1.8% and 6% of children, making it one of the most pervasive neurodevelopmental disorders (American Psychiatric Association, 2013).

Literature investigating the psychosocial consequences of poor motor skills has only recently experienced significant empirical development. Skinner and Piek (2001) indicated that children (8–10 years) and adolescents (12–14 years) with DCD reported lower self-worth and higher anxiety when compared to their non-DCD peers. In addition, adolescents with DCD reported higher anxiety than children with DCD, suggesting that these psychosocial consequences become more pronounced with age. In their sample of 47 children with DCD, Green et al. (2006) identified a significant proportion of participants were at risk of psychopathology. Children with both poor motor skills and emotional/behavioral difficulties (EBD) have also been identified as having more depressive symptoms and more problematic behaviors than EBD-only children (Heath et al., 2005). Such findings have stimulated further research in this area of investigation.

Within the literature, the etiology and implications of poor motor skills are often investigated from a diagnostic perspective. Analogous to Skinner and Piek (2001), studies often employ a diagnosis of DCD (or ‘probable-DCD’) to dichotomize their population into those with or without motor coordination problems, and examine differences between the groups. This approach is not without its limitations, but has proven useful within research seeking to investigate the psychosocial consequences of poor motor skills (when poor motor skills are operationalized within a clinical context). Results have indicated that DCD is associated with a range of psychosocial consequences, including less enjoyment in daily tasks (Bart et al., 2011), low self-esteem (Miyahara and Piek, 2006), less developed social support (Smyth and Anderson, 2000; Skinner and Piek, 2001), poor social skills (Kanioglou et al., 2005), social isolation (Smyth and Anderson, 2000), academic underachievement (Alloway, 2007), peer victimization/bullying (Campbell et al., 2012), decreased quality of life (Hill et al., 2011), and physical inactivity (Cairney et al., 2005). Each of these associated difficulties have important links with internalizing problems (anxiety and depression) in their own right. Furthermore, an individual with DCD could be exposed to any combination of these psychosocial consequences that may contribute to the onset and maintenance of internalizing difficulties, and also complicate intervention strategies.

There is consistent support for an association between DCD and internalizing problems from studies that vary in terms of both population and research design. Skinner and Piek (2001) employed a cross-sectional research design and found that children and adolescents with DCD had higher levels of anxiety than those without. The finding that the association between DCD and internalizing problems becomes more pronounced over time was limited by the cross-sectional design. This has since been supported longitudinally by Lingam et al. (2012). In their community sample of 6,902 children, the authors identified that a diagnosis of probable DCD at 7 years of age was associated with depressive symptoms and mental health difficulties at 10 years of age. Sigurdsson et al. (2002) also identified that poor motor skills in childhood were a risk factor for anxiety in adolescence. These findings provide preliminary suggestion that poor motor skills precede the development of internalizing problems. A monozygotic twin study by Piek et al. (2007) was able to account for genetic effects and shared environmental influences in their sample of 24 pairs of monozygotic twins discordant for probable DCD, whilst also controlling for the confounding influence of ADHD. It was found that twins with DCD demonstrated higher levels of depressive symptoms when compared to the unaffected co-twins. The authors suggested that the higher level of internalizing problems could be attributed to the unique environmental experiences of the twin with DCD, such as more negative peer interactions, academic underachievement, and negative self-perceptions (Piek et al., 2007). Such studies provide examples of the growing evidence to support an association between DCD and internalizing problems, although this area of investigation is currently lacking additional meta-analytic support.

Studies that employ the comparison of DCD (or probable DCD) groups to non-DCD groups have made important contributions to our understanding of the association between poor motor skills and internalizing problems. However, this approach does not take into account that motor skills are distributed dimensionally, rather than dichotomously, throughout the population. Subsequently, there has been an increase in studies that have enlisted community samples which reflect the broader spectrum of motor skills. Cross-sectional and longitudinal studies of community samples have indicated a negative association between motor skills and internalizing symptoms across the full spectrum of motor skills; better motor skills are associated with lower levels of internalizing symptomology (Piek et al., 2010; Rigoli et al., 2012; Wilson et al., 2013; Poole et al., 2015). These findings demonstrate that the psychosocial implications of motor skills are present across the full spectrum of ability, promoting further research in community samples. Therefore, this mini-review focuses on understanding the relationship between the full spectrum of motor skills (including DCD and non-DCD populations) and internalizing problems, and how these intermediary psychosocial issues may mediate this association.

Studies investigating the association between motor skills and internalizing problems have been largely empirical. While the association between motor skills and internalizing problems is well documented, this area is currently limited by a lack of theoretical development underlying the causal nature of this association (Cairney et al., 2013). In this mini-review we discuss the recently proposed Elaborated Environmental Stress Hypothesis by Cairney et al. (2013) that attempts to address this current limitation. A brief overview of the model is provided, before discussing the recent empirical studies evaluating this theoretically driven framework.

## The Elaborated Environmental Stress Hypothesis

**FIGURE 1:**
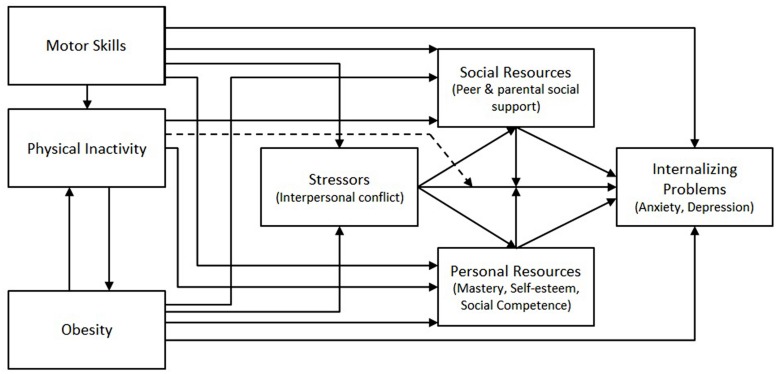
**An adapted Elaborated Environmental Stress Hypothesis.** This adapted version examines motor skills across the full spectrum of ability; the original Elaborated Environmental Stress Hypothesis described by Cairney et al. (2013) specifically focused on children with developmental coordination disorder (DCD).

The Elaborated Environmental Stress Hypothesis (Cairney et al., 2013) provides a conceptual model (see **Figure 1**) that allows for the testing of causal pathways from motor skills to internalizing problems. This recent framework expanded on the earlier work of Cairney et al. (2010). The Elaborated Environmental Stress Hypothesis illustrates the complex relationship between motor skills and internalizing problems and posits DCD as a primary stressor that exposes the individual to a range of secondary psychosocial stressors (e.g., peer conflict, low social support, poor academic performance, peer victimization, low self-esteem, low self-competence, physical inactivity, and obesity). It is hypothesized that the consistent exposure to these secondary stressors may then give rise to the onset and maintenance of internalizing problems through these potential mediating and moderating variables, whilst also acknowledging that the relationship between motor skills and internalizing problems is likely to be an interaction of both genetic and environmental factors. A more complete description of the model can be found in Cairney et al. (2013).

The Elaborated Environmental Stress Hypothesis was originally developed within the context of children with DCD, and how their poor motor skills may give rise to internalizing problems. However, there is evidence to support the application of the framework across the broader spectrum of motor skills. A negative linear association between motor skills, internalizing problems and other psychosocial variables described in the Elaborated Environmental Stress Hypothesis has been found in community samples (e.g., Wilson et al., 2013; Poole et al., 2015), suggesting that the psychosocial implications of motor skills extend beyond the DCD population. This negative linear association demonstrates that better motor skills are associated with decreased psychosocial problems. This provides important information for prevention and intervention strategies. For example, universal intervention programs that aim to promote motor skills may have psychosocial benefits for the wider population, rather than being limited to only those with DCD (e.g., Piek et al., 2015).

## Recent Developments

Cairney et al. (2013) provide a comprehensive description of the previous literature that contributed to the development of the Elaborated Environmental Stress Hypothesis. Therefore the present focus is on the recent developments that have occurred since the framework was published. However, where appropriate, some earlier literature is reflected upon in light of these more recent findings. At the time this mini-review was written, the Cairney et al. (2013) framework had been cited 22 times in peer-reviewed literature (*Google Scholar citations, 19 November 2015*). Some of this literature has reflected on the development of the model and its possible use in future studies (e.g., Missiuna and Campbell, 2014), while others have started to provide the empirical evaluation necessary to test this causal framework.

Illustrated in **Figure 1**, the Elaborated Environmental Stress Hypothesis highlights the complex causal network that describes how poor motor skills may lead to internalizing problems. Empirically evaluating this entire framework in a single study presents a range of methodological considerations that make such an approach difficult. These considerations include the response burden for participants, difficulties acquiring a large enough sample size to detect the likely small effects (Wilson et al., 2013), and the complexity of the analyses required to evaluate the full model. Consequently, the Elaborated Environmental Stress Hypothesis is more practically investigated through studies that evaluate a smaller combination of pathways embedded within the broader causal model. This has led to several studies that have since evaluated the various moderating/mediating variables specified by the framework (see **Table 1**).

**Table 1:** Summary of recent, peer-reviewed empirical support for the elaborated environmental stress hypothesis by Cairney et al. (2013).

Study	Research design	*N*	Sample type	Key supporting findings
Lingam et al., 2012	Longitudinal	6,902	Community; children (7–10 years)	Motor skills at 7 years of age predict mental health problems at 10 years of age. Poor social skills, peer victimization, self-esteem, and perceived scholastic competence mediate this relationship, in children with probable DCD.
Rigoli et al., 2012	Cross-sectional	93	Community; adolescents (12–16 years)	Self-perceptions mediate the association between motor skills and internalizing problems
Wilson et al., 2013	Cross-sectional	532	Community; children (4–6 years)	Social skills mediate the association between motor skills and internalizing problems
Viholainen et al., 2014	Cross-sectional	327	Community; adolescent females (12–16 years)	Self-concepts mediate the association between motor skills and internalizing problems
McIntyre et al., 2014	Intervention	35	Clinical; adolescents (13–17 years)	Intervention improving exercise in populations with low motor competence also improved their physical self-perceptions
Piek et al., 2015	Intervention	337^∗^	Community; children (4–6 years)	Interventions improving motor skills and participation improve prosocial behavior
Poole et al., 2015	Longitudinal	189^∗^	Community; lifespan (0–40 years)	Childhood motor difficulties predict internalizing problems in adulthood. Limited by retrospective report of childhood motor difficulties at age 29–35 years. Mental health problems measured at 8 years, 22–26 and 29–36 years only.

*N = Total number of participants. ^∗^Final sample at final follow-up periods.*

These studies have used a combination of different samples and research designs, and often provided support for the framework. Wilson et al. (2013) found the relationship between motor skills and internalizing problems in a community sample of 475 young children (4–6 years) to be mediated by social skills. Rigoli et al. (2012) enlisted a community sample of 93 adolescents (12–16 years). Their findings provided further support for the framework; self-perceptions were found to mediate the association between motor skills and internalizing problems. Similar findings have been replicated in a more recent study by Viholainen et al. (2014) with a community sample of 327 female adolescents (12–16 years). Self-concept, specifically related to school-related physical education, was found to mediate the relationship between motor skills and psychosocial wellbeing in this cohort. These results provide further support for this key pathway embedded within the framework.

Recent support for the Elaborated Environmental Stress Hypothesis extends beyond cross-sectional studies. Recent findings by Piek et al. (2015) conducted the first intervention study to evaluate the Elaborated Environmental Stress Hypothesis. The authors use 6 and 18-month follow up data of a 4–6 years old community population who participated in the randomized control trial (RCT) of the *Animal Fun* program (Wilson et al., 2013). The *Animal Fun* program is a 10-week school-based universal intervention program aimed at promoting motor development in 4–6 years old children. Findings from the RCT indicated the intervention group demonstrated significant improvement in prosocial behavior at 6-month follow-up, which remained at 18-month follow up. These results provide support for the pathways in the framework which suggest that interventions to improve motor skills and physical engagement will result in secondary improvements in psychosocial areas such as prosocial behavior. Similarly, Bart et al. (2009) found that intervention improving balance reduces anxiety and increased self-esteem in children with comorbid balance and anxiety disorders.

McIntyre et al. (2014) identified that participation in a 13-week physical activity intervention program for adolescents with poor motor skills resulted in an increase in physical self-perceptions, which is congruent with the pathways specified within the framework. A limitation of this study was that no measure of internalizing symptoms was included. However, previous studies have identified that increased physical self perceptions act as a protective factor against internalizing symptoms in similarly aged populations (Rigoli et al., 2012; Wilson et al., 2013; Viholainen et al., 2014).

Longitudinal evaluation of the Elaborated Environmental Stress Hypothesis is currently limited. However, recent longitudinal studies provide support for the causal relationship between motor skills and internalizing problems; this association underpins the entire framework. Lingam et al. (2012) used a community sample of 6,902 children and identified that motor skills difficulties at 7 years of age were associated with mental health difficulties (including internalizing problems) at 10 years of age; however, mental health difficulties were not measured at 7 years of age which limited the ability to conclude a causal relationship. The authors did identify several mediating variables between motor skills and mental health difficulties which were relevant to the Elaborated Environmental Stress Hypothesis, namely poor social skills, peer victimization, self-esteem, and perceived scholastic competence. More recently, Poole et al. (2015) published the results of a longitudinal study which included measures of motor skills and mental health. Their study provided an indication that childhood coordination problems were associated with internalizing problems in adulthood. However, childhood motor difficulties were reported retrospectively at age 29–35 years, relying on a subjective recount of motor skills. Similarly, mental health problems were only measured at age 8 (parent and teacher report) and self-report at age 22–26 and 29–36 years. Consequently, there is a need for more rigorous longitudinal investigation of motor skills and mental health.

These studies provide some indication that poor motor skills do precede the development of internalizing problems. In addition, they also emphasize that motor difficulties in early childhood are associated with psychosocial issues in later life. However, it is important to note that a current lack of longitudinal studies that consider shared risk factors for internalizing problems and motor skills such as infant emotional regulation and maternal stress limits the ability to make conclusions about the causal relationship between motor skills and internalizing problems as specified by the Elaborated Environmental Stress Hypothesis. Research by Hill and Brown (2013) has contributed qualitative support for the psychosocial impacts of DCD in adulthood. Within the recent developments pertaining to the Elaborated Environmental Stress Hypothesis, it was noted that several of these studies used community samples, moving beyond investigating the DCD population only. Each of these studies were able to provide support for different components of the overall framework. These findings promote the efficacy of the Elaborated Environmental Stress Hypothesis as a theoretical framework that is meaningful across the full spectrum of motor skills (and across the lifespan), rather than limited to DCD in childhood only.

While the framework provides a comprehensive description highlighting poor motor skills as a risk factor for internalizing problems, it is important to recognize the other risk factors that may contribute to the onset of anxiety and depression in childhood. Shaw et al. (1997) identified several risk factors during infancy that were related to the development of internalizing problems at pre-school age. Similarly, low socio-economic status and low birth weight/gestational age have also been identified as both risks for DCD and internalizing problems (Lingam et al., 2009). Such evidence provides a suggestion that these pre-disposing risk factors for internalizing problems may have similar repercussions for motor development. While the measurement of infant emotional regulation is notoriously difficult, further longitudinal investigations that consider the risk factors for internalizing factors and poor motor skills (e.g., maternal stress and infant emotional regulation) is necessary in order to achieve a better understanding of the association between internalizing problems and motor skills, and to evaluate the Elaborated Environmental Stress Hypothesis.

## Conclusion

This mini-review has provided an up-to-date appraisal of the Elaborated Environmental Stress Hypothesis by Cairney et al. (2013). The Elaborated Environmental Stress Hypothesis provides a framework to describe how poor motor skills may lead to the development of internalizing problems through a range of secondary psychosocial consequences. The ability to make accurate, coordinated, age-appropriate movements facilitates the ability to meet developmental milestones and foster opportunities for positive social engagement. Consequently, poor motor skills may increase psychosocial difficulties. It is important to recognize that the Elaborated Environmental Stress Hypothesis does not intend to provide a complete explanation of the etiology of internalizing problems, as psychopathology is multi-factorial. Rather, the purpose of this framework is to highlight motor skills as a potentially important factor to consider when understanding the onset of internalizing problems. There is a growing body of empirical support for the framework, using cross-sectional, intervention, and longitudinal research designs. The key finding from this mini-review is that the Elaborated Environmental Stress Hypothesis has utility beyond the DCD population. Studies enlisting community samples have provided evidence supporting the Elaborated Environmental Stress Hypothesis as a useful framework to understand the psychosocial implications of motor skills across the full spectrum of motor skills. This causal framework also provides a useful tool in the development of intervention strategies. Initiatives that aim to improve motor skills can be complemented by psychosocial components that focus on improving the secondary psychosocial stressors of poor motor skills. This has the capacity to buffer the impact of poor motor skills on internalizing problems. The negative linear association between motor skills and psychosocial issues provides support for universal motor skill interventions such as *Animal Fun*. Preliminary findings have shown that implementation of this movement program in children increased psychosocial wellbeing (Piek et al., 2015). Further empirical investigation of the Elaborated Environmental Stress Hypothesis is recommended, as not all of the causal pathways specified in the framework have been empirically tested, particularly using longitudinal designs. Similarly, there is an alternative argument to suggest that shared predisposing risk factors associated with internalizing problems may have similar implications for motor development (e.g., maternal stress, low socioeconomic status); longitudinal studies are required in order to address this argument. Future evaluations of the framework should identify how key pathways may differ across different contexts (e.g., age/gender), which could facilitate the development of more targeted psychosocial interventions. Future research should consider the model developmentally, in order to identify if these pathways differ across different developmental periods.

## Author Contributions

VM was the primary author of this study, and wrote the mini-review and received feedback from each of the other supervisors. DR, JC, LR, and JP were all supervisors as part of this PhD research topic. Each of the supervisors provided insight, expertise, and feedback on the paper. They provided several edits and proofreads throughout the refinement of the article.

## Conflict of Interest Statement

The authors declare that the research was conducted in the absence of any commercial or financial relationships that could be construed as a potential conflict of interest.
